# *Stem Cell Reviews and Reports*: Cancer Stem Cells and Aging Section

**DOI:** 10.1007/s12015-017-9727-3

**Published:** 2017-02-10

**Authors:** Peter Quesenberry

**Affiliations:** Rhode Island Hospital, Warren Alpert Medical School of Brown University, Providence, RI 02903 USA

The cellular origins of cancer have long been debated. Work indicating the existence of a rare stem cell for acute leukemia was followed by a number of reports of relatively rare stem cells for a number of cancers. Other work has challenged the concept of cancer stem cells being a rare repopulating cell for cancer or providing a promising target for chemotherapy or immunotherapy. A balanced view here may be that there may be a continuum of “stemness” with early cancers having rare stem cells while with progression , i.e. most solid tumors, most of the cancer cells are in fact stem cells. In this latter case there would be no advantage to defining these cells. Thus far therapeutic application of cancer stem cell directed therapy has been disappointing. There is also longstanding and abundant information on the role of microenvironment in carcinogenesis. This bring up the issues of whether cancer cell mutations or micro environmental influences are predominant in carcinogenesis.

The area of aging and stem cells interfaces with carcinogenesis as the latter progressively increases with age. The increasing clonal nature of blood stem cells with aging is pertinent here. Many studies have suggested interesting changes in hematopoietic stem cell numbers and function but have not taken into account the issues of the stem cell continuum and baseline stem cell hematopoiesis. Major issue are whether aging effects can be reversed or manipulated and what role the systemic effects of aging have on neoplastic hematopoietic diseases.

The section on Cancer stem cells and Aging provides a forum for the exchange of ideas and results from areas pertaining to cancer stem cells and carcinogenesis in general and the impact of aging on stem cell number and function and neoplastic potential. We will emphasize

- The definition of cancer stem cells;
- The characterization as to total phenotype of putative cancer stem cells;
- The model systems available for study;
- The regulation of putative cancer stem cells:-the therapeutic implication of cancer stem cells;
- The changes in hematopoiesis and hematopoietic stem cells with aging;
- The nature of clonal hematopoiesis with aging;
- The study of different stem cell models with aging;
- The capacity to modulate aging effects of hematopoiesis;
- The implications for hematologic malignancies with regard to aging hematopoietic stem cells;
- The therapeutic implications of aging hematopoietic stem cell populations

We look forward to receiving your high-quality submissions to our journal and also welcome your feedback and opinions on the new structure and other future changes.

